# Proton pump inhibitor *versus* potassium‐competitive acid blocker in gastroesophageal reflux disease

**DOI:** 10.1002/jgh3.13104

**Published:** 2024-06-02

**Authors:** Tadayuki Oshima

**Affiliations:** ^1^ Department of Gastroenterology Okazaki City Medical Association Public Health Center Okazaki Aichi Japan; ^2^ Department of Gastroenterology and Metabolism Nagoya City University Graduate School of Medical Science Nagoya Aichi Japan

Gastroesophageal reflux disease (GERD) is a highly prevalent disorder with a significant impact on patients' quality of life (QOL), and its global prevalence is increasing. Therapy goals for GERD encompass symptom resolution, healing of esophageal inflammation, and prevention of complications. Healing of esophageal erosions, in particular, has been traditionally emphasized as an objective measure and primary endpoint in clinical trials. In this context, the findings presented by Simadibrate *et al*.^1^ in this journal issue support the potential superiority of potassium‐competitive acid blockers (P‐CABs) as a maintenance therapy for GERD. P‐CABs have been developed to meet the unmet needs of conventional proton pump inhibitors (PPIs), providing rapid, long‐lasting, and reversible inhibition of the proton pump (H^+^, K^+^ ATPase α subunit).^2^ Recent other meta‐analyses and network meta‐analyses have evaluated the efficacy of vonoprazan, keverprazan, and tegoprazan for the maintenance therapy of GERD. Vonoprazan, especially, has demonstrated superior efficacy over conventional PPIs in maintaining GERD treatment, particularly in cases of severe reflux esophagitis.^3^, ^4^

While mucosal healing remains crucial in GERD maintenance, the importance of heartburn‐free days should not be underestimated. However, only one study has shown a significant increase in 24‐h heartburn‐free days during maintenance, and one‐fifth of GERD patients still experienced symptoms despite maintenance treatment with vonoprazan 20 mg or lansoprazole 15 mg.^5^ Consequently, further investigations are necessary to definitively determine the superior efficacy of P‐CABs over conventional PPIs. Moreover, there is still room for devising treatments to improve GERD symptoms, beyond simply suppressing acid secretion.

Furthermore, it is essential to consider the minimal effective dose in treatment to mitigate the risk of side effects. The safety profile of P‐CABs remains uncertain, particularly regarding the contentious issue of gastric cancer development and alterations in gut microbiota. Additionally, data on the long‐term safety of keverprazan and tegoprazan are scarce. Given the incomplete assessment of gastric acid suppression levels with these medications, caution is warranted in interpreting the available data, especially regarding effective dosages and the comparative characteristics among P‐CABs.

When assessing the efficacy of GERD treatment, the varying degrees of gastric acid suppression among P‐CABs with different doses emerge as a crucial consideration. Given the observed differences in *Helicobacter pylori* eradication rates and gastrin levels among P‐CABs, it becomes essential to evaluate the extent of gastric acid suppression and potential disparate effects among these medications. Notably, with increasing doses of vonoprazan, the occurrence of nocturnal acid breakthrough (NAB) is markedly reduced,^6^ in contrast to conventional PPIs that exhibit limited nocturnal acid suppression. These distinctions likely contribute to the increased rate of *H. pylori* eradication seen in regimens including vonoprazan.^7^

In the context of initial GERD treatment, reflux symptoms often persist inadequately after the initial dose of PPIs in approximately two‐thirds of patients due to the slow onset of action, with around half of patients still experiencing symptoms even after 3 days of treatment. Vonoprazan has demonstrated rapid acid suppression compared with lansoprazole and has shown superior efficacy in achieving complete heartburn relief, particularly within the first week of therapy, in patients with erosive esophagitis.^8^ Moreover, vonoprazan has been found to provide more effective nighttime heartburn relief than lansoprazole in this patient population and has been shown to improve sleep quality within 1 week, a benefit not observed with lansoprazole. Recent research has indicated that tegoprazan exhibits more rapid and potent nighttime acid suppression compared with vonoprazan or esomeprazole when administered nocturnally.^9^ A network meta‐analysis has further highlighted that vonoprazan is either equally or more effective than conventional PPIs in resolving heartburn on both Day 1 and Day 7 in patients with erosive esophagitis.^10^ Consequently, vonoprazan may be considered as a first‐line therapy to alleviate symptoms and enhance patients' QOL.

The treatment efficacy of P‐CABs for GERD requires meticulous assessment. Given the potential variation in acid suppressive effects and approved doses among P‐CABs, direct comparison of treatment effects among them is challenging. Therefore, individual evaluation of each P‐CAB is essential for future analyses, rather than considering them collectively as a group.
